# Supplementary material for: DMRforPairs: identifying Differentially Methylated Regions between unique samples using array based methylation profiles
Source: BMC Bioinformatics. 2014 May 15;15:141. doi: 10.1186/1471-2105-15-141 (PMC4046028; doi:10.1186/1471-2105-15-141)
Supplement: Additional file 2 — DMRforPairs output for the comparison of A431-MCF7 and NA17018-NA17105. Please start from the HTML files in each folder. Available via the BMC Bioinformatics website. [file 1471-2105-15-141-S2.zip › 1394847754114233_MOESM2_ESM/A431_MCF7/figures/16675.pdf]

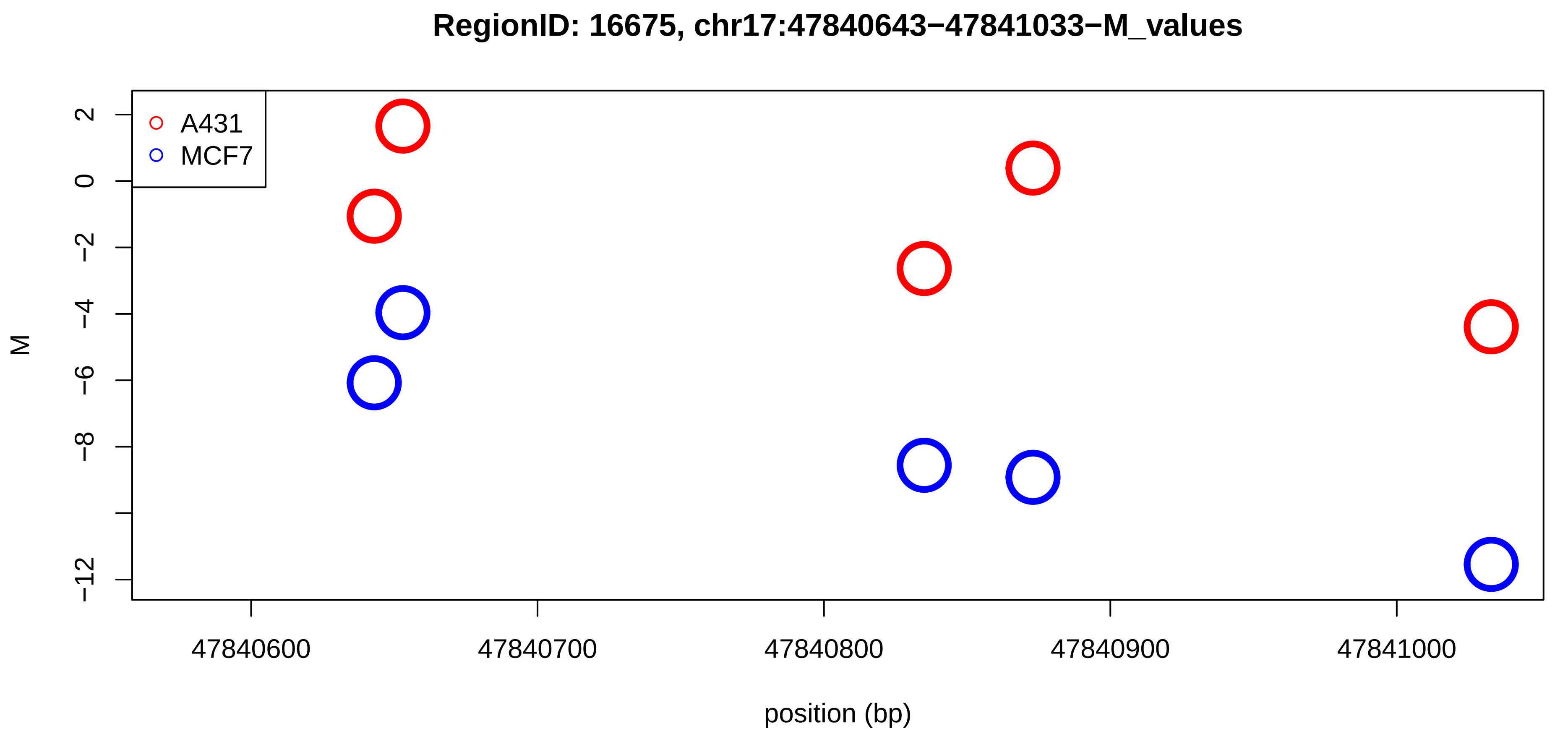
RegionID: 16675, chr17:47840643−47841033−M_values
A431
MCF7
M
2
0
−2
−4
−6
−8
−12
47840600
47840700
47840800
47840900
47841000
position (bp)

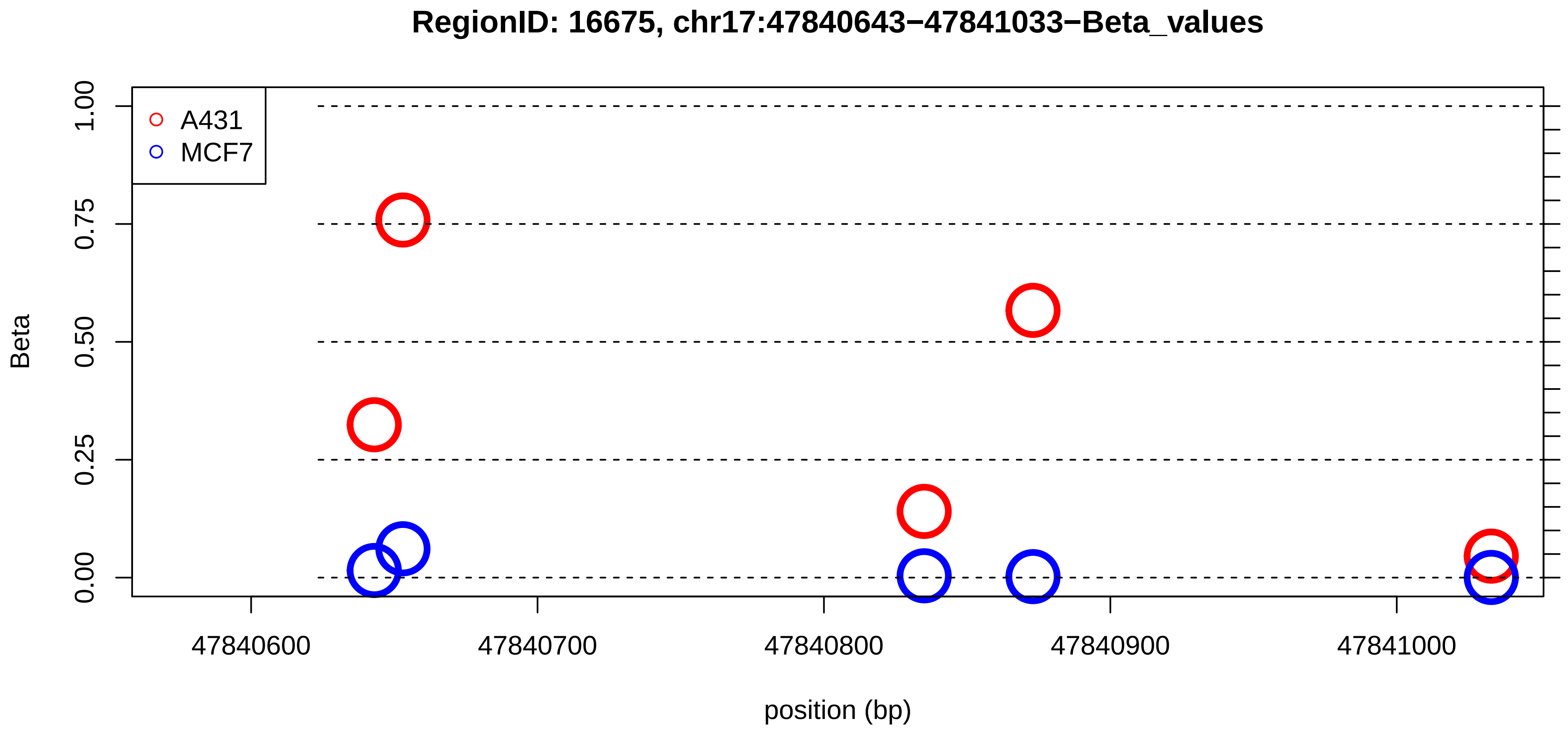
RegionID: 16675, chr17:47840643−47841033−Beta_values
A431
MCF7
Beta
1.00
0.75
0.50
0.25
0.00
47840600
47840700
47840800
47840900
47841000
position (bp)
